# Re: A novel approach to treatment of priapism refractory to non-surgical methods: A single-case experience. By Nassib Abou Heidar, Jad A. Degheili, Gerges Bustros, Wassim Wazzan and Muhammad Bulbul

**DOI:** 10.1080/2090598X.2019.1640931

**Published:** 2019-07-31

**Authors:** Ahmed El-Assmy

**Affiliations:** Urology and Nephrology Center, Urology Department, Mansoura University, Mansoura, Egypt, a_assmy@yahoo.com

Dear Editor,

Ischaemic priapism (IP) is a true urological emergency. Once IP is diagnosed, the first line of treatment entails ejaculation, physical exercise and cold fomentation to stimulate the noradrenergic system, leading to smooth muscle contraction and detumescence. If a conservative approach fails, corporal aspiration followed by an injection of α-adrenergic agonist into the corpus cavernosum is considered a very valuable option. If IP lasts >24–36 h, these patients are very unlikely to respond due to smooth muscle necrosis [1].

If corporal blood aspiration and injections of α-adrenergic agonists fail, the second-line treatment consists of penile shunt surgery. In the event of an IP lasting >36 h, shunt surgery is unlikely to be effective. In these types of cases, early penile prosthesis implantation can be considered [1].

Abou Heidar et al. [2] report a case of a 58-year-old man presenting with 24-h IP refractory to cavernous injections of phenylephrine and a percutaneous T-shunt procedure. After refusal of surgery, a continuous cavernous infusion of phenylephrine for 8 h overnight under monitoring was performed, with resolution of the erection and no complications.

I have great concerns about such a management strategy. Firstly, the continuous infusion regimen for 8 h is too long a time, with a consequently higher dose of phenylephrine being absorbed. Systemic complications (e.g., hypertension, arrhythmia and septicaemia) and local complications (e.g., necrosis and infection) of prolonged infusion could occur, and both systemic and local complications are very serious. Secondly, hypertensive patients and those with ischaemic heart disease would be at more risk of life-threating cardiovascular events.

Trying this approach successfully in one patient does not justify offering it as a new management option. Further studies are needed to test the safety profile of continuous penile infusion with phenylephrine. Until this happens, I do not encourage the use of this method by general urologists for the treatment of refractory IP.
